# Real-world study of the effectiveness of BBIBP-CorV (Sinopharm) COVID-19 vaccine in the Kingdom of Morocco

**DOI:** 10.1186/s12889-022-14016-9

**Published:** 2022-08-20

**Authors:** Yaowen Zhang, Jihane Belayachi, Yunkai Yang, Qiang Fu, Lance Rodewald, Hongling Li, Bing Yan, Ying Wang, Yanna Shen, Qian Yang, Weiyun Mu, Rong Tang, Chen Su, Tianfang Xu, Majdouline Obtel, Abdelkader Mhayi, Rachid Razine, Redouane Abouqal, Yuntao Zhang, Xiaoming Yang

**Affiliations:** 1 China National Biotec Group Company Limited, B2 Shuangqiao Rd, Chaoyang District, Beijing, P.R. China; 2 Acute Medical Unit, Ibn Sina University Hospital, Rabat, Morocco; 3 Laboratory of Biostatistics, Clinical, and Epidemiological Research, Department of Public Health, Faculty of Medicine and Pharmacy, Mohammed V University in Rabat, Rabat, Morocco; 4 China Sinopharm International Corporation, Beijing, P.R. China; 5 Chinese Center for Disease Control and Prevention, National Immunization Program, Beijing, P.R. China; 6 Laboratory of Community Health (Public Health, Preventive Medicine and Hygiene), Department of Public Health, Faculty of Medicine and Pharmacy, Mohammed V University in Rabat, Rabat, Morocco; 7 Department of Informatics, Ministry of Health and Social Protection, Rabat, Morocco

**Keywords:** COVID-19, Vaccine, Effectiveness, BBIBP-CorV

## Abstract

**Background:**

The Kingdom of Morocco approved BBIBP-CorV (Sinopharm) COVID-19 vaccine for emergency use on 22 January 2021 in a two-dose, three-to-four-week interval schedule. We conducted a retrospective cohort study to determine real-world BBIBP-CorV vaccine effectiveness (VE) against serious or critical hospitalization of individuals RT-PCR-positive for SARS-CoV-2 during the first five months of BBIBP-CorV use in Morocco.

**Methods:**

The study was conducted among adults 18–99 years old who were tested by RT-PCR for SARS-CoV-2 infection between 1 February and 30 June 2021. RT-PCR results were individually linked with outcomes from the COVID-19 severe or critical hospitalization dataset and with vaccination histories from the national vaccination registration system. Individuals with partial vaccination (< 2 weeks after dose two) or in receipt of any other COVID-19 vaccine were excluded. Unadjusted and adjusted VE estimates against hospitalization for serious or critical illness were made by comparing two-dose vaccinated and unvaccinated individuals in logistic regression models, calculated as (1-odds ratio) * 100%.

**Results:**

There were 348,190 individuals able to be matched across the three databases. Among these, 140,892 were fully vaccinated, 206,149 were unvaccinated, and 1,149 received homologous BBIBP-CorV booster doses. Unadjusted, full-series, unboosted BBIBP-CorV VE against hospitalization for serious or critical illness was 90.2% (95%CI: 87.8—92.0%). Full-series, unboosted VE, adjusted for age, sex, and calendar day of RT-PCR test, was 88.5% (95%CI: 85.8—90.7%). Calendar day- and sex-adjusted VE was 96.4% (95%CI: 94.6—97.6%) for individuals < 60 years, and was 53.3% (95%CI: 39.6—63.9%) for individuals 60 years and older. There were no serious or critical illnesses among BBIBP-CorV-boosted individuals.

**Conclusions:**

Effectiveness of Sinopharm’s BBIBP-CorV was consistent with phase III clinical trial results. Two doses of BBIBP-CorV was highly protective against COVID-19-associated serious or critical hospitalization in working-age adults under real-world conditions and moderately effective in older adults. Booster dose vaccination was associated with complete protection, regardless of age, although only a small proportion of subjects received booster doses.

## Background

Coronavirus disease 2019 (COVID-19) is a public health emergency of international concern. As of March 2022, the World Health Organization (WHO) has received reports of more than 475 million cases and 6 million COVID-19 deaths [1]. The first case of COVID-19 in the Kingdom Morocco was reported to WHO on 2 March 2020. As of 8 February 2022, there have been 1,147,243 COVID-19 cases (3.2% of total population) and 15,593 COVID-19 associated deaths (0.04%) reported by Morocco to WHO [1]. There have been two complete epidemic waves in Morocco: the first started early November 2020, and the second started in August 2021. In January 2022, case counts rose again, signaling onset of a third wave. In April 2021, more than 90% of severe acute respiratory syndrome coronavirus 2 (SARS-CoV-2) isolates were the Alpha variant, with the rest being Beta and Gamma variants. In August 2021, during the second wave, 80% were Delta and 20% were Alpha. In January 2022, during the third wave, 90% of isolates have been Omicron, with the remainder being Delta [2].

COVID-19 vaccines were first introduced to Morocco on 28 January 2021 and recommended for individuals 18 years and above. Thus far, there have been five COVID-19 vaccines in widespread use in Morocco: inactivated virus vaccine (Beijing CNBG’s BBIBP-CorV), virus vectored vaccines (AstraZeneca’s Vaxzevria and Gamaleya’s Gam-COVID-Vac), mRNA vaccine (Pfizer-BioNTech’s Comirnaty), and modified virus vector vaccine (Janssen’s Ad26.COV 2-S). In February 2021, 7% of the population received one or more doses of COVID-19 vaccine, and by June 2021 26% had at least their first dose. As of 12 January 2022, there have been 51,321,365 doses of COVID-19 vaccines administered to 24,701,243 people; 66.14% of the total population has had one or more doses, and 61.83% of the population has been fully vaccinated [3]; 72% of the COVID-19 vaccines used were BBIBP-CorV.

BBIBP-CorV is a whole-virus, inactivated, alum-adjuvanted vaccine; its safety and immunogenicity were demonstrated in pre-clinical and phase I and II clinical trials [4–6]. Efficacy of BBIBP-CorV for preventing symptomatic infection was 78.1% in a phase III study conducted in United Arab Emirates, Bahrain, Egypt, and Jordan [7]. WHO approved BBIBP-CorV for emergency use listing on 5 May 2021 [8]; Morocco approved BBIBP-CorV for emergency use on 22 January 2021 [9], and was one of the first countries to approve the vaccine. According to the Morocco Department of Health, citizens and foreigners residing in Morocco could be vaccinated with BBIBP-CorV vaccine upon appointment. Priority groups for vaccination were healthcare personnel, front-line workers, seniors, and patients with chronic diseases. Full primary vaccination with BBIBP-CorV requires two intramuscular injections separated by an inter-dose interval of 21 to 28 days [10].

The phase III clinical trials of BBIBP-CorV had too few severe/critical cases to provide reliable efficacy estimates against the most severe outcomes [7]. Real-world studies with larger sample sizes are needed for such assessments. The protective effectiveness of BBIBP-CorV against serious or critical hospitalization associated with COVID-19 had not been assessed previously in Morocco. Pfizer-BioNTech’s BNT162b2, Moderna’s mRNA-1273, AstraZeneca’s AZD1222, and BBIBP-CorV have been widely used in many countries, but compared with these other vaccines, there have been the fewest studies of BBIB-CorV [11].

We report a real-world study in this period to estimate the effectiveness of BBIBP-CorV in the Kingdom of Morocco using official surveillance, testing, and vaccination data during the vaccine’s first five months of use.

## Methods

### Study design

We conducted a retrospective cohort study between 1 February and 30 June 2021 to evaluate vaccine effectiveness (VE) of BBIBP-CorV against serious or critical COVID-19-associated hospitalization among Morocco residents aged 18–99 who were tested by reverse transcription polymerase chain reaction (RT-PCR) for SARS-CoV-2 infection.

### Data sources

Study data were obtained from three databases, with individual-level data linked by national identification number. Vaccination data, including vaccination date, and vaccine type and dose were obtained from the National Vaccination Registry (NVR), which is an electronic health record system that records complete vaccination histories.

The SARS-CoV-2 testing strategy in Morocco promoted RT-PCR testing for all individuals including people with and without symptoms, close contacts of cases, and travelers at no charge to the public. RT-PCR results were reported to E-labs, which is a national COVID-19 laboratory network for diagnostic specimens tested by RT-PCR in Morocco. Antigen test results were not recorded in E-labs, nor were only clinically-confirmed cases. Individuals tested by RT-PCR were potential subjects for this study. We had access to all E-labs test results.

Clinical data on severity of COVID-19-associated hospitalization were recorded in a public health surveillance datasets made available for this study.

### Outcomes and vaccination status

In Morocco, all patients hospitalized for any severe illness were screened for COVID-19. A COVID-19 associated hospitalization for serious or critical illness was defined any hospitalization for severe/critical illness of an individual with SARS-CoV-2 RT-PCR positivity, regardless of whether hospitalization was a direct consequence of COVID-19 infection. Thus, the outcome for VE analysis was all-cause COVID-19 hospitalization.

Individuals were classified as fully vaccinated if at least 14 days had passed since the administration of a second dose of BBIBP-CorV vaccine. An unvaccinated, control population consisted of individuals who had not received any doses of any COVID-19 vaccine. Individuals who received three doses of BBIBP-CorV were categorized as boosted.

### Statistical analysis

Means and standard deviations (SD) were used to describe continuous variables; categorical variables were expressed as frequencies and percentages. We used logistic regression to estimate odds ratios (OR) and accompanying 95% confidence intervals (CI). We defined fully vaccinated status as receipt of 2 doses of BBIBP-CorV 2 or more weeks before date of testing. Booster-vaccinated status was receipt of 3 doses 2 or more weeks before date of testing. Not vaccinated status was the reference exposure. Unadjusted VE and adjusted VE (adjusting for age, sex, and calendar day of RT-PCR test) against serious or critical hospitalization were calculated as (1-OR)*100%. All data analyses were performed with SAS software (version 9.4, SAS Institute Inc., Cary, NC, USA).

## Results

**Fig. 1:**
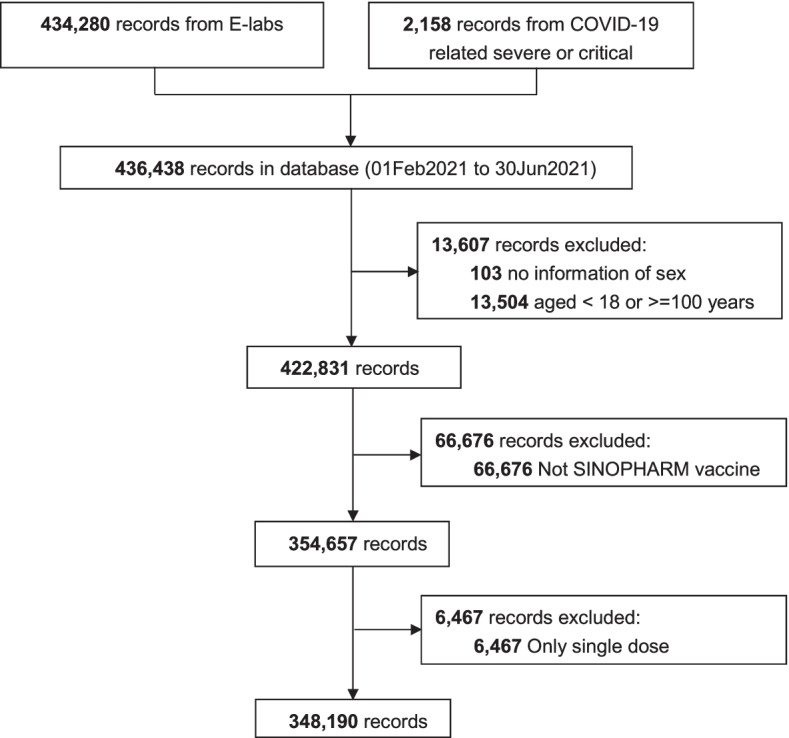
Flow diagram of the study population with eligibility criteria, exclusions, and matching methodology

Figure 1 shows the study flow diagram and inclusions and exclusions of potential subjects. From 1 February 2021 to 30 June 2021, a total of 436,438 records from the COVID-19 related severe or critical hospitalization dataset and E-labs were able to be matched with vaccination histories in the NVR. After exclusions for missing data on sex or age, administration of a vaccine other than BBIBP-CorV, and partial vaccination, 348,190 records remained and comprised the analytic dataset.

Table 1 shows the vaccination status of subjects by sex and age group. All subjects in the study were RT-PCR positive. Among subjects, there were more men than women (57.3 and 42.7%) and more younger people than older people – for example, 29.9% were 18–29 years old and 9.9% were 60 years or older. There were 206,149 individuals (59.2% of subjects) completely unvaccinated, 140,892 (40.5%) individuals fully vaccinated with two doses of BBIBP-CorV, and 1,149 (0.3%) individuals who received booster doses.

**Table 1:** Characteristics of the study subjects by BBIBP-CorV vaccination status, Morocco, 1 February 2021 through 30 June 2021

	Not Vaccinated ***N* = 206,149**	Fully Vaccinated ***N* = 140,892**	Booster-vaccinated ***N* = 1,149**	Total ***N* = 348,190**
Sex, n (%)				
Male	116,816 (56.7)	81,580 (57.9)	1,024 (89.1)	199,420 (57.3)
Female	89,333 (43.3)	59,312 (42.1)	125 (10.9)	148,770 (42.7)
Age (Years)				
Mean (SD)	40.7 (15.1)	37.9 (13.5)	45.1 (15.8)	39.6 (14.6)
Age group (Years), n (%)				
18–29	56,553 (27.4)	47,313 (33.6)	201 (17.5)	104,067 (29.9)
30–39	53,558 (26.0)	35,226 (25.0)	336 (29.2)	89,120 (25.6)
40–49	41,896 (20.3)	29,515 (20.9)	195 (17.0)	71,606 (20.5)
50–59	27,576 (13.4)	21,263 (15.1)	151 (13.1)	48,990 (14.1)
> = 60	26,566 (12.9)	7,575 (5.4)	266 (23.2)	34,407 (9.9)

N (%) refer to numbers and percentages of each group

Abbreviations: *SD* Standard deviation

Table 2 shows serious or critical hospitalization broken down by BBIBP**-**CorV vaccination status, sex, and age group. Among the 348,190 study subjects, 1,436 (0.41%) had serious/critical hospitalization while SARS-CoV-2 RT-PCR positive. Among hospitalized subjects, 52.1% were male and 61.1% were less than 60 years old. Compared to unvaccinated individuals, fewer individuals who received BBIBP-CorV primary-series vaccination were hospitalized with severe/critical COVID-19 (0.65 vs 0.06%, *p* < 0.001). No individuals who received BBIBP-CorV booster doses were hospitalized for severe/critical COVID-19.

**Table 2:** Proportions of serious or critical hospitalization by vaccination status, stratified by sex and age group

	Serious/critical hospitalization N (%)	No serious/criticalhospitalization N (%)	*p*-value
**Main analysis**			
Not Vaccinated	1,345 (0.65)	204,804 (99.35)	< .001
Fully Vaccinated	91 (0.06)	140,801 (99.94)	
Booster-vaccinated	0 (0)	1,149 (100)	
**Subgroup analysis**			
**Male**			
Not Vaccinated	688 (0.59)	116,128 (99.41)	< .001
Fully Vaccinated	60 (0.07)	81,520 (99.93)	
Booster-vaccinated	0 (0)	1,024 (100)	
**Female**			
Not Vaccinated	657 (0.74)	88,676 (99.26)	< .001
Fully Vaccinated	31 (0.05)	59,281 (99.95)	
Booster-vaccinated	0 (0)	125 (100)	
**18–29 years**			
Not Vaccinated	65 (0.11)	56,488 (99.89)	< .001
Fully Vaccinated	0 (0)	47,313 (100)	
Booster-vaccinated	0 (0)	201 (100)	
**30–39 years**			
Not Vaccinated	182 (0.34)	53,376 (99.66)	< .001
Fully Vaccinated	0 (0)	35,226 (100)	
Booster-vaccinated	0 (0)	336 (100)	
**40–49 years**			
Not Vaccinated	254 (0.61)	41,642 (99.39)	< .001
Fully Vaccinated	6 (0.02)	29,509 (99.98)	
Booster-vaccinated	0 (0)	195 (100)	
**50–59 years**			
Not Vaccinated	353 (1.28)	27,223 (98.70)	< .001
Fully Vaccinated	18 (0.08)	21,245 (99.90)	
Booster-vaccinated	0 (0)	151 (100)	
**< 60 years**			
Not Vaccinated	854 (0.48)	178,729 (99.52)	< .001
Fully Vaccinated	24 (0.02)	133,293 (99.98)	
Booster-vaccinated	0 (0)	883 (100)	
**> = 60 years**			
Not Vaccinated	491 (1.85)	26,075 (98.15)	< .001
Fully Vaccinated	67 (0.88)	7,508 (99.12)	
Booster-vaccinated	0 (0)	266 (100)	

N (%) refer to numbers and percentages of each group. *P***-**values were calculated using Chi**-**Squared test

Table 3 shows unadjusted and adjusted vaccine effectiveness by sex and age group. Unadjusted, unboosted full-series BBIBP-CorV vaccine effectiveness against serious or critical hospitalization was 90.2% (95%CI: 87.8—92.0%). After adjusting for age, sex, and calendar day of RT-PCR test in the logistic regression model, adjusted full-series, unboosted VE against serious or critical hospitalization was 88.5% (95%CI: 85.8—90.7%). Adjusted full-series, unboosted VE was higher among females (92.6%, 95%CI: 89.4—94.9%) than males (83.8%, 95%CI: 78.9—87.6%), and higher among individuals < 60 years (96.4%, 95%CI: 94.6—97.6%) than individuals 60 years and older (53.3%, 95%CI: 39.6—63.9%).

**Table 3:** Unadjusted and adjusted two-dose, unboosted BBIBP-CorV vaccine effectiveness against serious or critical hospitalization among 18–99-year-olds in Morocco

	Unadjusted VE % (95%CI)^**a**^	Adjusted VE % (95%CI)
**Main analysis**		
Not Vaccinated	Ref	Ref
Fully Vaccinated	90.2 (87.8–92.0)	88.5 (85.8–90.7)
**Subgroup analysis**		
**Male**		
Not Vaccinated	Ref	Ref
Fully Vaccinated	87.6 (83.8–90.5)	83.8 (78.9–87.6)
**Female**		
Not Vaccinated	Ref	Ref
Fully Vaccinated	92.9 (89.9–95.1)	92.6 (89.4–94.9)
**18–29 years**		
Not Vaccinated	Ref	Ref
Fully Vaccinated	100 (NA-NA)	100 (NA-NA)^b^
**30–39 years**		
Not Vaccinated	Ref	Ref
Fully Vaccinated	100 (NA-NA)	100 (NA-NA)^b^
**40–49 years**		
Not Vaccinated	Ref	Ref
Fully Vaccinated	96.3 (92.5–98.5)	97.0 (93.3–98.7)
**50–59 years**		
Not Vaccinated	Ref	Ref
Fully Vaccinated	93.5 (89.5–95.9)	93.9 (90.2–96.2)
** < 60 years**		
Not Vaccinated	Ref	Ref
Fully Vaccinated	96.2 (94.3–97.5)	96.4 (94.6–97.6)
** > = 60 years**		
Not Vaccinated	Ref	Ref
Fully Vaccinated	52.6 (38.7–63.3)	53.3 (39.6–63.9)

*Abbreviations*: *VE* Vaccine effectiveness, *CI* Confidence interval, *NA* value is not available, *Ref* Reference

^a^Unadjusted VE and adjusted VE (adjusting for age, sex, and calendar day of RT-PCR test) were calculated as (1-odds ratio) * 100% from the logistic regression model

^b^Confidence interval could not be estimated using logistic regression because of zero events in the fully vaccinated group

## Discussion

Our study provided real-world evidence of the effectiveness of BBIBP-CorV vaccine under conditions of widespread use in Morocco and showed that among all age groups, primary series vaccination with BBIBP-CorV was 89% effective against serious or critical COVID-19 hospitalization. Among working-age adults, 2-dose VE against serious/critical COVID-19 hospitalization was 96%, and among older adults, 2-dose VE was 53%. Adjusted VE was slightly higher among females than males. There were no serious/critical COVID-19 hospitalizations for any booster-dose recipients, regardless of age. Our findings are consistent with phase III clinical trials and other real-world studies of this vaccine, and support continued use of BBIBP-CorV and emphasize the importance of a booster dose.

The outcome for our study was all-cause severe/critical hospitalization with RT-PCR positivity for SARS-CoV-2. This outcome is a key driver of health resource utilization in the COVID-19 pandemic because of its association with intensive care unit admission and ventilator use. Thus, reduction in severe/critical hospitalization is one of the most important purposes of COVID-19 vaccination.

In the phase III clinical trial of BBIBP-CorV conducted in the United Arab Emirates, we obtained a VE result of 78.1% against symptomatic wild-type COVID-19 [7]. In Argentina, two-dose vaccine effectiveness (VE) against COVID-19 related mortality was 84% among those aged ≥ 60 years [12], with the prevalent variants being Alpha, Gamma, and Lambda [13]. In a nation-wide observational study in Hungary with 895,000 adult BIBP-CorV recipients, two-dose VE was 69% against infection and 88% against COVID-19 related mortality, with the predominant variant being Alpha [14]. In a cohort study conducted in the United Arab Emirates among individuals 15 years and older, two-dose BBIBP-CorV VE was 79.8% against hospitalization and 92.2% for preventing COVID-19-related critical care admission when compared to a non-vaccinated group, with the predominant variants being Alpha and Beta [15].

Pfizer-BioNTech’s BNT162b2 mRNA vaccine and Moderna’s mRNA-1273 vaccine have been well studied around the world. Full-series vaccination with BNT162b2 or mRNA-1273 VE against severe COVID-19 is 90% or more in the general population. Two studies in Israel showed BNT162b2 VE against severe disease to be 92% and 97.5% [16, 17], with the main epidemic strain of Alpha. In Alpha-endemic areas of the United States, BNT162b2 or mRNA-1273 VE against ICU admission was 90% [18]. In Canada, BNT162b2 VE was 98% against hospital admission or death [19], during a period with Alpha, Beta, and Gamma variants circulating. Our results showed that BBIBP-CorV had similar VE against severe disease in the overall population.

Our study found that VE against all-cause serious or critical COVID-19 hospitalization was lower among older adults compared with working age adults: 96.4% (95%CI: 94.6—97.6%) for individuals < 60 years and 53.3% (95%CI: 39.6—63.9%) for individuals 60 years and older. Effectiveness of Pfizer, Moderna, and AstraZeneca COVID-19 vaccines for prevention of severe disease or hospitalization varied from 70 to 90% among older adults [19–23]. A study in the Netherlands [24] showed that mRNA-1273 effectiveness against ICU admissions declined with age and was 34% among individuals ≥ 70 years of age. Moustsen-Helms [25] and Yelin [26] showed results that are similar to ours. There are several possible reasons for decreased effectiveness with older age. Elderly people have more comorbidities [27], and vaccine-induced immune responses in the elderly are slower and SARS-CoV-2 neutralization is lower [28]. Since older adults were vaccinated before younger adults, their vaccine protection had more time to wane between vaccination and infection. As with other COVID-19 vaccines, waning protection requires booster vaccination to sustain protection.

Our study has several limitations. First, there were no analyses of virus strains, and VEs against specific SARS-CoV-2 variants could not be estimated. According to the WHO reports, more than 90% of SARS-CoV-2 isolates were Alpha variants in Morocco in April 2021 [2], thus the VE estimates are mainly against the Alpha strain. Second, we lacked data on comorbidities and other characteristics of the study population that could influence VE estimates, precluding controlling for potential confounding factors other than age, sex, and calendar date of RT-PCR testing. Third, we did not have data on reasons for severe/critical hospitalization. As a result, our study could not distinguish hospitalization caused by SARS-CoV-2 infection from hospitalization coincidental to SARS-CoV-2 infection. Because older individuals are more likely than younger adults to be hospitalized for any reason, our all-cause VE results among the elderly are likely an underestimate of VE against SARS-CoV-2-caused COVID-19 severe/critical hospitalization. Fourth, because elderly adults were recommended earlier than younger adults for COVID-19 vaccination, the time between vaccination and infection would be relatively longer, which may have confounded our VE estimate among elderly adults through waning immunity. This would bias the VE estimate among elderly downward. Finally, the timing of the study was such that too few individuals had received booster doses, precluding robust analysis of BBIBP-CorV booster dose VE.

## Conclusions

Effectiveness of Sinopharm’s BBIBP-CorV was consistent with phase III clinical trial results. Two doses of BBIBP-CorV was highly protective against COVID-19-associated serious or critical hospitalization in working-age adults under real-world conditions and moderately effective in older adults. Booster dose vaccination was associated with complete protection, regardless of age, although only a small proportion of subjects received booster doses.

## Data Availability

The data are available from the Morocco’s National Control Commission for the Protection of Personal Data (CNDP), which were used under license for the current study, but restrictions apply to the availability and therefore are not publicly available. However, data are available from the authors upon reasonable request and with permission of said registry holders and Ethics Board.

## Abbreviations

COVID-19: Coronavirus disease 2019
NVR: National Vaccination Registry
RT-PCR: Reverse transcription polymerase chain reaction
SARS-CoV-2: Severe acute respiratory syndrome coronavirus 2
SD: Standard deviations
VE: Vaccine effectiveness
WHO: World Health Organization
